# Occurrence of Toxic Cyanobacterial Blooms in Rio de la Plata Estuary, Argentina: Field Study and Data Analysis

**DOI:** 10.1155/2012/373618

**Published:** 2012-02-22

**Authors:** L. Giannuzzi, G. Carvajal, M. G. Corradini, C. Araujo Andrade, R. Echenique, D. Andrinolo

**Affiliations:** ^1^Cátedra de Toxicología, Facultad de Ciencias Exactas, Universidad Nacional de La Plata, Calle 47 y 115 (1900), La Plata, Argentina; ^2^Instituto de Tecnología, Facultad de Ingeniería y Ciencias Exactas, Universidad Argentina de la Empresa, Lima 717, C1073AAO Buenos Aires, Argentina; ^3^Unidad Académica de Física de la Universidad Autónoma de Zacatecas, Calzada Solidaridad S/N, Esquina con Paseo de la Bufa, 98060 Zacatecas, ZAC, Mexico; ^4^Departamento Científico de Ficología, Facultad de Ciencias Naturales y Museo, Universidad Nacional de La Plata, Calle 47 y 115 (1900), La Plata, Argentina

## Abstract

Water samples were collected during 3 years (2004–2007) at three sampling sites in the Rio de la Plata estuary. Thirteen biological, physical, and chemical parameters were determined on the water samples. The presence of microcystin-LR in the reservoir samples, and also in domestic water samples, was confirmed and quantified. Microcystin-LR concentration ranged between 0.02 and 8.6 **μ**g.L^−1^. Principal components analysis was used to identify the factors promoting cyanobacteria growth. The proliferation of cyanobacteria was accompanied by the presence of high total and fecal coliforms bacteria (>1500 MNP/100 mL), temperature ≥25°C, and total phosphorus content ≥1.24 mg*·*L^−1^. The observed fluctuating patterns of *Microcystis aeruginosa*, total coliforms, and Microcystin-LR were also described by probabilistic models based on the log-normal and extreme value distributions. The sampling sites were compared in terms of the distribution parameters and the probability of observing high concentrations for *Microcystis aeruginosa*, total coliforms, and microcystin-LR concentration.

## 1. Introduction

The presence of cyanobacteria and their toxic metabolites, cyanotoxins, in water reservoirs normally used as domestic water supplies is increasingly being reported. Among cyanotoxins, microcystins (MCs) are considered one of the most dangerous groups. MCs are known to be potent hepatotoxins [1] and tumor promoters [2].

Field studies in South Africa [3] and Canada [4] have shown that environmental factors are associated with toxin concentration during cyanobacterial blooms. Additionally Chorus [5] reported the influence of environmental factors on MC levels.

The assessment of water quality in a reservoir usually involves monitoring multiple parameters. The sampling procedure is performed at predetermined intervals and many points of interest (sampling points) are included. A complex data matrix is frequently needed to evaluate water quality [6]. Furthermore, in river monitoring, one is frequently faced with the problem of determining whether a variation in the concentration of measured parameters can be attributed to pollution (man-made, spatial) or to natural (temporal, climatic) changes in the aquatic systems' hydrology. As a result of the latter, one has also to establish which parameters are the most significant to describe such spatial and temporal variations, the pollution sources, and so forth. By identifying relevant contributions one can characterize a point of interest, for example, recreational park, water intake, and evaluate its risk in terms of the prevalence of conditions associated to the high or low probability of an undesirable event. To obtain additional information from the collected data, it is also desirable to explore the possibility of translating the irregular fluctuation pattern of each factor into a set of probabilities of future high counts as was proposed for other water resources [7]. In principle, such probabilities can be used to quantify the water's contamination level and pattern, compare the behavior at different sampling sites, and also as a tool to evaluate the potential efficacy of corrective and/or preventive measures.

**Figure 1:**
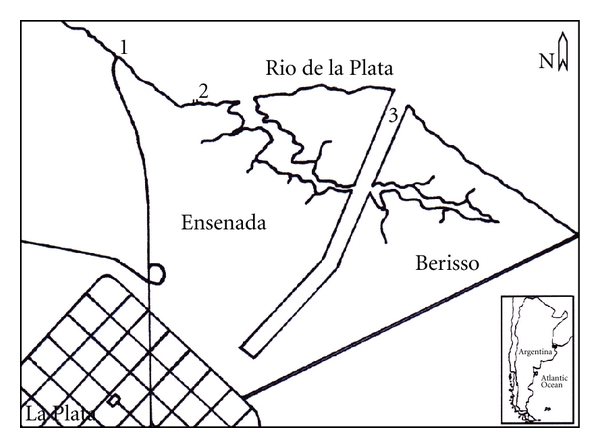
Estuary area sampling sites. Station 1: main intake of La Plata City water treatment plant, Station 2: the harbor access, and Station 3: a recreational river channel.

The Rio de la Plata estuary is located at the margins of Argentina and Uruguay. This water body constitutes a valuable water resource for the province of Buenos Aires, Argentina, which is a vast area of 3.000.000 km^2^ with more than 20 million inhabitants (see Figure 1). Besides the agricultural, industrial, and recreational uses of this water body, it is also the main source of drinking water for large cities located on its coastline, such as Buenos Aires and Montevideo. Since the 1950s, pollution has severely increased by the progressive settlement of industry and urban development in the coastal region of Rio de la Plata. Leaching out of fertilizers from agricultural areas and urban runoff has contributed to increase eutrophication of the estuarine region.

During the austral summer 1999, short-term blooms of *Microcystis aeruginosa* were observed at two locations on the Uruguayan coast of the Rio de la Plata near the city of Colonia [8]. The hepatotoxic heptapeptides microcystins have been so far the only identified toxins within this estuary [8]. Toxic cyanobacterial blooms on the Argentinean margin of the Rio de la Plata as well as the identity of the toxins present in the toxic blooms have been described previously by Andriolo et al. [9]. Consequently, the occurrence of potentially hepatotoxic cyanobacterial blooms presents a significant health hazard to humans, livestock, and wildlife. Cyanobacterial and fecal contamination of water sources has always been a major safety concern and a factor in determining the need of a treatment.

The distribution and toxicity of coliforms and cyanobacteria blooms along reservoirs are usually heterogeneous; thus, human exposure to toxins is not easily established from routine sampling [10].

In the present work, samples were collected during 3 years (2004–2007) at different sampling points located at a channel of La Plata Harbor within the Rio de la Plata estuary. Thirteen parameters were determined for each sample. This complex data matrix was treated using principal components analysis (PCA) in order to reduce the data dimensionality without loss of valuable information. This procedure allowed us to characterize both temporal and spatial variations in the water quality as well as to identify different variation patterns associated with either seasonal variations or pollution sources.

A major difficulty in assessing the microbial quality of water in streams and reservoirs, apart from the issues related to sampling and culturing the organisms of interest, is the irregular nature of the records. The collected data usually fluctuate widely and are punctuated by aperiodic outbursts of unpredictable magnitude and duration. Models based on population dynamics [11–13] and chaos theory [14, 15] have been used to describe the oscillating populations of coliforms. Their effectiveness, however, is limited when applied to natural habitats due to relatively low sampling frequency that does not permit to follow the population's evolution in sufficient detail. Also, the microbial population's response to local environmental changes and its relation to accidental contamination cannot be revealed by periodic counts if these are too widely spaced. This is particularly true for fecal organisms and cyanobacteria, whose source or origin in an open system such as a flowing river cannot be fully traced. Nevertheless, in many places, because of logistic considerations, this is the only feasible option to monitor the water quality, and the result is records of very limited value for formulating a population.

The objectives of the present study were to monitor the presence of cyanobacteria and their toxic metabolites in Rio de la Plata river and in domestic water samples of La Plata city, to identify the effect of biological and environmental factors on their occurrence, and to test the possibility of estimating future cyanobacteria, total coliforms, and toxin high concentrations from the irregular fluctuating records of these counts.

## 2. Material and Methods

### 2.1. Sampling Points

Samples were collected at a channel within La Plata Harbor. Three sampling locations were selected on the south coast of the La Plata River, 60 Km south-west of the city of Buenos Aires, Argentina. The first sampling site, Station 1 (34°49′13′′S and 57°57′49′′W) is located where the main intake of La Plata City water treatment plants takes place and is representative of the raw drinking water quality. Station 2 is placed at the harbor access (34°49′51′S and 57°56′50′′W), about 10 km upstream from Stations 1 and 3 (34°50′04′′S and 57°52′41′′W). The latter location corresponds to a recreation river entrance. Both Stations (1 and 3) are multipurpose sites, since they are used for recreational and sport activities, fishing, water supply, and even serve as an international port and a mobile station (see Figure 1).

The parameters to be monitored were selected based on the recommendations of the Global Environmental Monitoring System, United Nations Environmental Program GEMS/Water UNEP program [16] and the World Health Organization [17] as well as the spatial and seasonal changes in the water quality over the studied river section.

Sample collection, including the selection of adequate containers, stabilization, and transport to the laboratory as well as storage were performed according to the GEMS/Water Operational Guide [16] and as described by Pesce and Wunderlin [18]. Samples were taken at least 40 cm under the water surface and whenever possible, at the middle of the stream. The depth was selected to avoid excessive contributions of run off material and to have a representative sample of the intake of the water treatment plant. Although vertical gradients of cyanobacteria have been observed in several studies, a significant difference was only observed at locations deeper than the one selected in this study [19, 20]. Water sampling at each point (Stations 1–3) was performed with a monthly or fortnightly periodicity.

Sample collection was cancelled on rainy days, and on these occasions it was rescheduled at least 72 h after the rain had stopped, in order to allow for the river to return to its regular flow condition and avoid excessive mixing of the water column.

Surface water was also collected on two occasions about 2 days after massive episodes of marine life death in the region (on December 2004 and January 2006) were observed.

### 2.2. Phytoplankton Analysis

Phytoplankton samples were obtained using a 30 *μ*m mesh plankton net. An aliquot of these samples was analyzed “*in vivo*” using a Wild M20 microscope. The optic microscope was furnished with a drawing and photographic camera. Once studied, samples were fixed with a 50% Transeau solution. Additional samples were obtained using a Van Dorn bottle and were used for quantification purposes. The samples used during quantification were fixed in situ with a 1% lugol solution for their subsequent analysis with a reverted microscope, following the Utermöhl methodology [21]. The samples were stored and transported to the laboratory on ice chests. Qualitative and quantitative phytoplankton determinations and principal nutrient (phosphorous and nitrogen) analysis were performed in duplicates.

### 2.3. Monitored Parameters and Analytical Methods

The selection of parameters to be monitored at all sampling sites was based on an evaluation of the nutrients recommended by WHO [16, 17]. Standard analytical methods developed and/or compiled by the American Public Health Association (1998) [22] were used for each chosen parameter (the method number for each determination is provided between parentheses). The measured parameters included chlorophyll-a (10200-H-spectrophotometric), fecal coliforms (9221 E), total coliforms (9221B), iron (3500-Fe D), nitrates (4500- NO3_ E), nitrites (4500- NO2_ B), orthophosphate phosphorus (4500-P over samples previously treated by acid hydrolysis and digested with persulphate), pH (4500-H_ B, field measured); temperature (2550-B, field measured), conductivity (2510 A field measured). All the determinations were performed in duplicate.

### 2.4. Microcystin Detection

Microcystin determination was performed on samples obtained at the three samples sites within the river. In order to test the presence of this toxin in household potable water additional samples were taken at a selected point of the domiciliary distribution network and analyzed.

To detect MCs, each water sample (500 mL) was subjected to 3 freezing-thawing cycles then filtered and finally applied to a preactivated C-18 solid-phase extraction cartridge Sep-Pak C18 ODS (Waters, Milford, MA, USA) which was previously conditioned with methanol (10 mL) and 5% acetic acid (10 mL). The cartridge was washed with 10 mL of 10, 20, and 30% aqueous methanol and toxins were eluted with 3 mL of pure methanol. The eluate was evaporated to dryness under medium vacuum (40°C, 0.3 Torr) and resuspended in 500 mL of methanol prior to chromatographic analysis of MCs. The quantitative chromatographic analysis of MCs was performed using high performance liquid chromatography (HPLC) with a photodiode array detector (Shimadzu LC- 20 A, SPD-M20 A, Shimadzu Scientific Instruments, Columbia, MD, USA) and a C18 column Thermo (5 *μ*m pore, 150 × 4.60 mm). The column was equilibrated with a mixture composed by 65% of solution A (0.05% (v/v) trifluoroacetic acid (TFA) in water) and 35% of solution B (0.05% (v/v) TFA in acetonitrile). The mobile phase consisted of a discontinuous gradient of A and B solutions. The flow rate was set to 1.0 mL.min^−1^. Standards of MC-RR, MC-YR and MC-LR were purchased from Sigma (St. Louis, MO, USA). MCs were identified on the basis of their UV spectra and retention time. UV detection was performed at 238 nm.

## 3. Statistical analysis

### 3.1. Principal Component Analysis

Principal components analysis (PCA) was used to elucidate the contribution of environmental parameters to cyanobacterial and cyanotoxin presence. Prior to applying this methodology, the data were mean-centered and scaled for each attribute. In this preprocessing stage the average value was subtracted from each variable which ensured that all results are interpretable in terms of variation around the mean. The scaling factor used for the data analysis was the inverse of the standard deviation [23].

All the environmental factors were included in this study and the records were transformed by applying log_10_(*x* + 1) to guarantee that principal components are independent. The data were analyzed using a Multivariate Statistical Package (MVSP, Kovach Computing Services, Wales, UK), and PCA was carried out by The Unscrambler v.9.8 software from CAMO Inc. (CAMO Software AS, Oslo, Norway).

### 3.2. Estimation of Future High Counts or Concentrations

Records of *Microcystis aeruginosa*, total coliforms, and microcystin concentrations obtained during the 2004–2006 period were characterized using several distribution functions. The records successive counts independence was tested using an autocorrelation function as reported by Peleg et al. [24]. Six symmetric and asymmetric distribution functions were examined (normal, log normal, Weibull, gamma, extreme value, and Laplace). The parameters of each distribution were estimated using two methods, namely, methods of moments (MM) and maximum likelihood estimation (MLE) readily available in MATHEMATICA (Wolfram, Champaign, IL), the software used throughout this section of the study. The applicability of the distribution functions was assessed in terms of their probability plots and their adequacy to fit the overall shape of the record's distribution including its tail. Since high counts and concentrations are correlated with higher risks for a population, the inadequate characterization of this section of the records might underestimate the probability of dangerous events. The performance of the distribution functions on estimating future high biological counts was tested by comparing the predicted frequency exceeding different cutoff values calculated from the records obtained in 2006 with those actually observed in 2007. Although we have focused on calculating the probability of high microbial counts and toxin concentration, this methodology could also be used to estimate the probability of low counts just by selecting lower cutoff values. Additionally, the sampling sites were compared based on the parameters of the relevant distribution functions and the probability of observing values above the cutoff values set by international organizations for each parameter of interest.

## 4. Results and Discussion

Records of total cyanobacteria and *Microcystis aeruginosa*, as well as the observed biological, physical, and chemical parameters, for the three selected sampling points, namely, 1, 2, and 3, are shown in Table 1.

Throughout the studied period the phytoplankton in the coastal waters of the Rio de la Plata estuary was dominated by *Microcystis aeruginosa*, which accounted for 97% of the total observed. Total phytoplankton counts ranged from 150 to 458400 cells·mL^−1^ and those of *M. aeruginosa* exhibited values from 0 to 458400 cells·mL^−1^.

Total and fecal coliforms were present in high concentrations in all water samples, the values obtained ranged from 1500 to 4600 MNP·100 mL^−1^. Seasonality did not play an important role on their concentrations. The minimum (11°C) and maximum (30°C) water temperatures were observed during winter and summer, respectively; however the temperature records exhibit large fluctuations during the observed period of time.

Water conductivity minimum value was observed in November (summer) while pH exhibited lower values during winter. The observed pH throughout this study was between 7.4 and 10.2.

Along with nitrogen and phosphorus content, iron concentration is one of the factors most likely to limit cyanobacteria growth in water ecosystems. Several studies indicate that iron content influences cyanotoxin production; however, the results are controversial [25–27]. Utkilen and Gjolme [28, 29] found a decrease in toxicity determined as the ratio of toxin to protein in a continuous culture of *Microcystis aeruginosa* when the iron concentration in the inflow medium was reduced from 10 to 0.3 *μ*M of FeCl_3_. Although the river samples' iron content was slightly higher during the peak months of the summer, high iron concentration values were neither limited to this period nor directly correlated to high cyanobacteria counts.

The literature related to the effects of nitrogen and phosphorus concentration on cellular microcystin content is also contradictory [23–29]. The observed phosphorus, nitrate, and nitrite concentrations of the water samples do not exhibit a clear pattern in relation to seasonality or cyanobacterial blooms. Although their presence always correlates to the observation of microcystin in the water samples, no conclusion about the effect of different phosphorus and nitrogen levels and microcystin concentration can be drawn from the data. It should be taken into account that the Rio de la Plata basin is the second largest in South America and as such it receives contributions of two main rivers (i.e., Parana and Paraguay) as well as several streams. Each contributor may affect the water flow pattern and the mineral load at the point of observation due to different agricultural crops and fertilization cycles throughout the seasons which may difficult the analysis.

The concentration of chlorophyll-a in water samples provides a reasonable estimate of algal biomass. The international guidelines for safe practice in managing recreational waters [17] has linked short-term adverse health outcomes, for example, skin irritation, low frequency of gastrointestinal illness, and potential for long-term illness to concentrations above 50 *μ*g·L^−1^. The chlorophyll-a concentration in the collected water samples always exceeded this value with a minimum of 12.0 mg·L^−1^ and a maximum 108 mg·L^−1^.

We confirmed the presence of microcystin-LR (MC-LR) in 90% of the samples that were positive for microcystins. MC-LR concentration at the different sampling sites showed values between 0.02 and 8.6 *μ*g·L^−1^. It should be noticed that only one peak was observed in the chromatograms with the same retention time of MC-LR. A typical elution profile of the water sample revealed a peak corresponding to microcystin-LR at 8.2 minutes retention time, and the typical microcystin absorption spectrum for this peak was also observed.

It is believed that MC is released from cyanobacteria after cell lysis, whereas only negligible amounts of toxins apparently are released from healthy cells [30]. However, a high concentration of soluble MC could be the result of rapid lysis of cyanobacteria, a probable situation during algaecide treatment of lakes [10], or through accumulation and subsequent lysis of cyanobacterial cells on filters in drinking-water treatment [31].

The World Health Organization (WHO) has established a provisional guide value of 1 *μ*g·L^−1^ for MC concentration in drinking water [32]. In order to establish the prevalence of this toxin in domestic samples, an additional sampling point within the potable water distribution system was selected and MR-LR was determined following the same procedure described for the river samples. The presence of microcystins in drinking water was detected in 10 out of 13 samples at values from <0.1–7.8 *μ*g·L^−1^. Based on this observation we can infer that microcystins released by *M. aeruginosa*, which can develop under eutrophic conditions in the Rio de la Plata estuary, reach the drinking water network, and attain concentrations that exceed the safe limit of 1 *μ*g·L^−1^ recommended by WHO [32]. Water treatment in the province of Buenos Aires, Argentina, comprises only the following steps: coagulation, sedimentation, filtration (sand filter), and chlorination. An activated carbon step, which can adsorb and eliminate the toxin when cyanobacterial blooms or cyanotoxins levels similar to the ones reported in this study are observed, is seldom applied.

## 5. Statistical Analysis

### 5.1. Principal Components Analysis (PCA)

To evaluate the changes in water quality that promote the occurrence of cyanobacteria, we used principal components analysis (PCA). It was performed to determine correlations between measured parameters of water samples and the presence of cyanobacterial blooms collected during the same period. PCA is a multivariate technique that operates in an unsupervised manner (each number of the groups under study is not known *a priori*) and it is used to analyze the inherent structure of the data.

PCA was applied independently to each sampling point (1–3) in order to identify groups correlated with the main influential factors that affect water quality and seasonality.

**Station 1**

**Figure 2:**
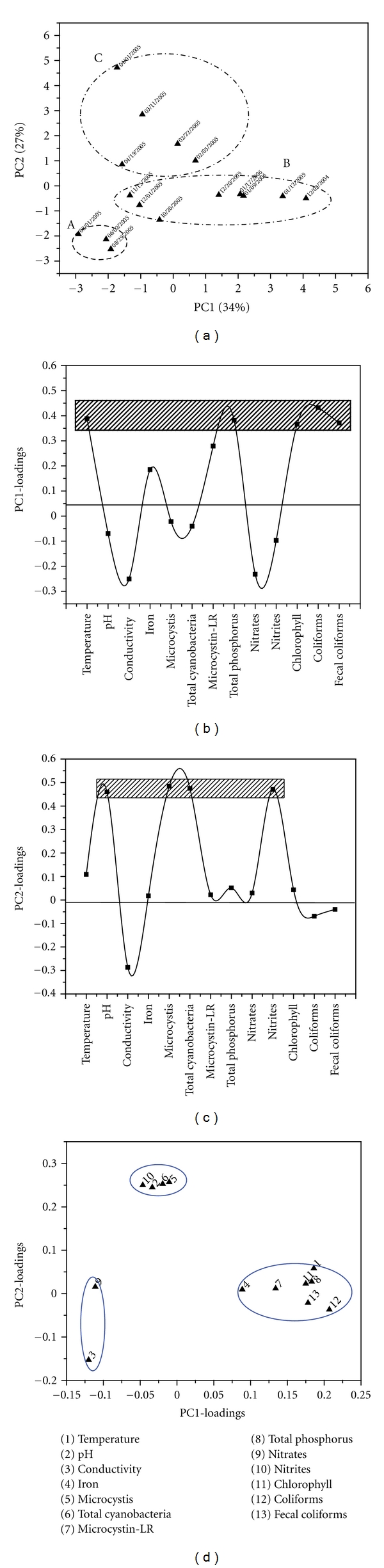
PCA results obtained for samples recollected from sampling point 1. (a) Two-dimensional PCA-scores plot for PC2 versus PC1, (b) and (c) correspond to one-dimensional loadings plots of PC1 and PC2, respectively, and (d) two-dimensional loadings plot.

The results obtained from PCA show that the first principal component PC1 explains 34% of total variance of the data and the PC2 explains 27% of it. In the scores plot of PC2 *versus* PC1, three clusters can be identified along PC2 axis. The groups A, B, and C correspond to samples collected on dates for the period of June to August, October to January and February to April, respectively (see Figure 2(a)).

Figure 2(b) shows that the main influential variables for the “objects” distribution along PC1-scores axis in the scores plot are temperature, and total phosphorus content, chlorophyll-a concentration, and total and fecal coliforms counts.

Water samples belonging to group A (i.e., 08/01/2005) differ in these variables from those at the end of group B (i.e., 12/03/2004).

As shown in Figure 2(c) the main influential variables for the three groups identified along the PC2-axis in the PCA-scores plot are pH, *M. aeruginosa, *and total cyanobacteria cell counts and nitrite concentration. Three attributes related to intervariable relationships (variable similarities) can be identified in Figure 2(d). *M. aeruginosa* and total cyanobacteria cell counts, nitrite concentration, and pH exhibit a strong relationship among them and also influence the separation of the three groups along PC2, as identified in the PCA scores plot.

**Station 2**

**Figure 3:**
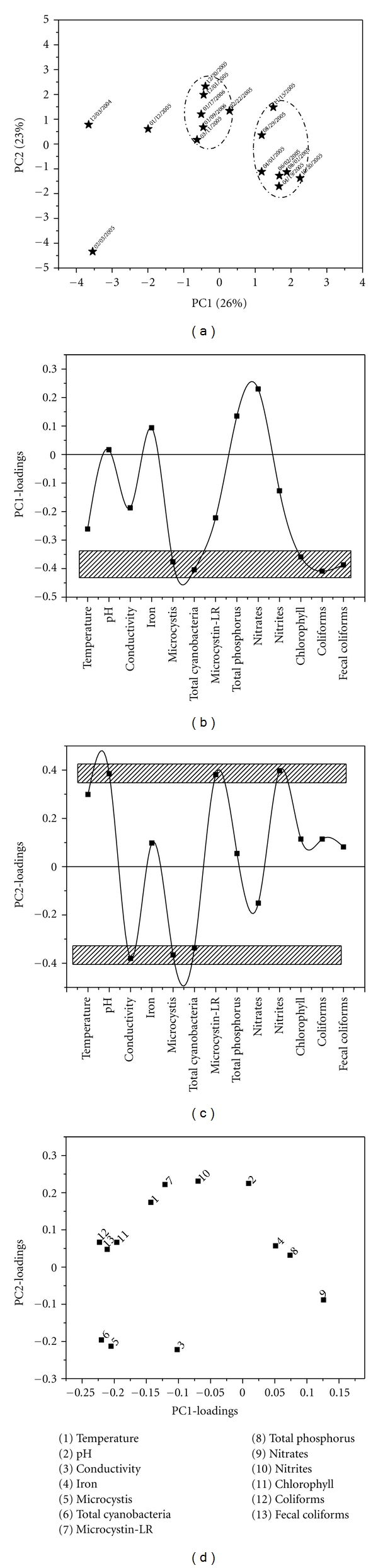
PCA results obtained for samples recollected from sampling point 2. (a) Two-dimensional PCA-scores plot for PC2 versus PC1, (b) and (c) correspond to one-dimensional loadings plots of PC1 and PC2, respectively, and (d) two-dimensional loadings plot.

26%, 23%, and 16% of total data variance can be explained by PC1, PC2, and PC3, respectively. In Figure 3(a) it is possible to identify two clear groups along the PC1-axis when the PCA-scores are plotted for PC2 versus PC1. On the other side, if the objects are projected over PC2-axis, it is also possible to recognize two distinct groups. To interpret this objects distribution, the loadings plots for PC1 and PC2 must be analyzed. The most influential variables (*M. aeruginosa*, total cyanobacteria cells, total and fecal coliforms counts, and chlorophyll-a content), related to the distribution of objects in the PCA scores plot, are showed within the rectangle in Figure 3(b). According to the score plots the distribution of the “objects” along PC1-axis is highly influenced and/or correlated with the presence, variation, or absence of these five attributes, whereas along the PC2-axis the distribution of the “objects” is influenced by the highest loading values (positive or negative), which in this case are pH, conductivity, *M. aeruginosa* and total cyanobacteria cell counts, and toxin and nitrite concentrations (see rectangles in Figure 3(c)). The two-dimensional loading plot (Figure 3(d)) can help us to identify intervariable relationships. *Microcystis aeruginosa*, chlorophyll-a, and total and fecal coliforms concentrations are highly related among them in the PC1-axis, but not in PC2. On the other hand, in the PC2-axis it is possible to observe the lack of correlation between the attributes conductivity and nitrite concentration.

**Station 3**

**Figure 4:**
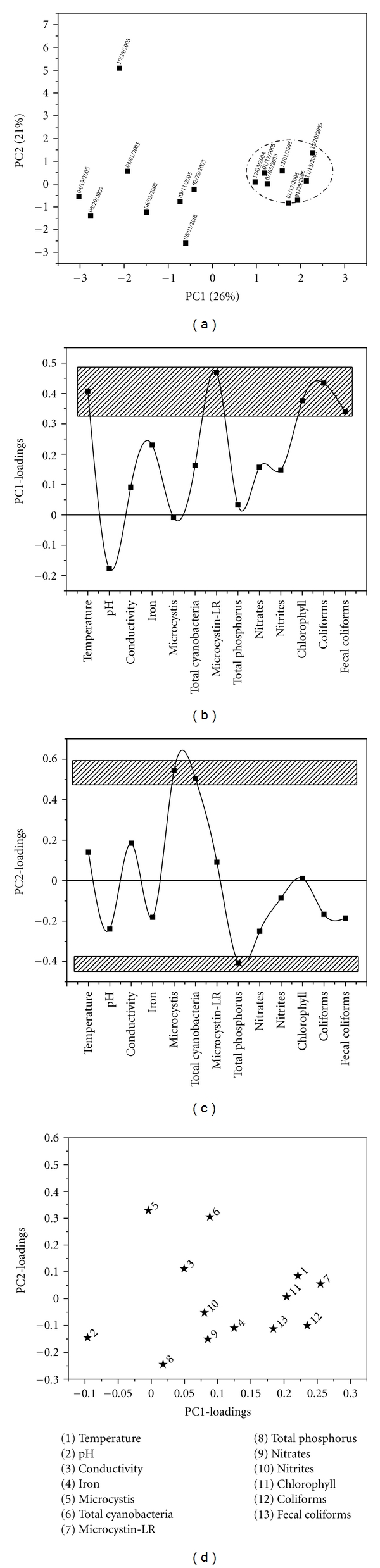
PCA results obtained for samples recollected from sampling point 3. (a) Two-dimensional PCA-scores plot for PC2 versus PC1, (b) and (c) correspond to one-dimensional loadings plots of PC1 and PC2, respectively, and (d) two-dimensional loadings plot.

It is possible to identify a clear group along the PC1-axis in Figure 4(a) (enclosed within the ellipse). If the objects are projected over PC2-axis, it is possible to recognize that object “10/20/2005” is different from the rest, it should be noticed that this object corresponds to the occurrence of rare event; the occurrence of a severe cyanobacterial bloom. The most influential attributes (temperature, toxin and chlorophyll-a concentrations, and total and fecal coliforms counts) related to the distribution of the objects in the PCA scores plot are showed within the rectangle in Figure 4(b).  Figure 4(c) shows the influential variables related with the distribution of the “objects” along the PC2-axis in the PCA-scores plot which is influenced and/or correlated to *M. aeruginosa*, total cells, total phosphorous, which are framed into the rectangles.

Wunderlin et al. [33] conducted a study where an array of different techniques including PCA analysis was used to evaluate spatial and temporal changes in the Suquía River water quality. This work involved monitoring 22 attributes at three sampling sites during two years. As a result of the analysis, the studied variables were classified in groups based on the observed changes and the effect of each group on overall water quality was assessed. Microcystin concentrations under different environmental conditions showed that the dominance of toxic strains could be favored at higher water temperatures (>23°C) and that high of ammonia-nitrogen (>36 *μ*M) and iron (>2 *μ*M) concentrations adversely affected cyanobacterial growth.

In the current study, the principal component analysis revealed that at the three sampling sites coliform, cyanobacterial, and *Microcystis aeruginosa *counts, microcystin concentration, temperature, and phosphorous content were the most relevant attributes in PC1. In regards to PC2, nitrite concentration and pH played an important role, and to a lesser extent* Microcystis aeruginosa and* cyanobacteria cell counts as well as microcystin concentration. Temperature records higher than 25°C favor proliferation of cyanobacterial blooms. This effect was most prevalent at two sampling sites (Stations 1 and 3). Paerl et al. [34] reported that excessive nitrogen loading in addition to phosphorus presence could be identified in many cases as a key culprit in eutrophication and cyanobacterial bloom's expansion, which explains the relevance of these conditions in the three sample sites.

### 5.2. Estimation of Microbial Counts and Toxin Concentrations above Specific Values

Although the principal component analysis performed on the recorded data identified the most relevant attributes that affect water quality in the Rio de la Plata estuary, this procedure does not allow for the estimation of the occurrence of future cyanobacterial blooms or future microcystin production above the guidelines suggested by WHO [32]. To this end a probabilistic approach as reported by Peleg et al. [7, 35–37] was pursued to complement the previous analysis.

**Figure 5:**
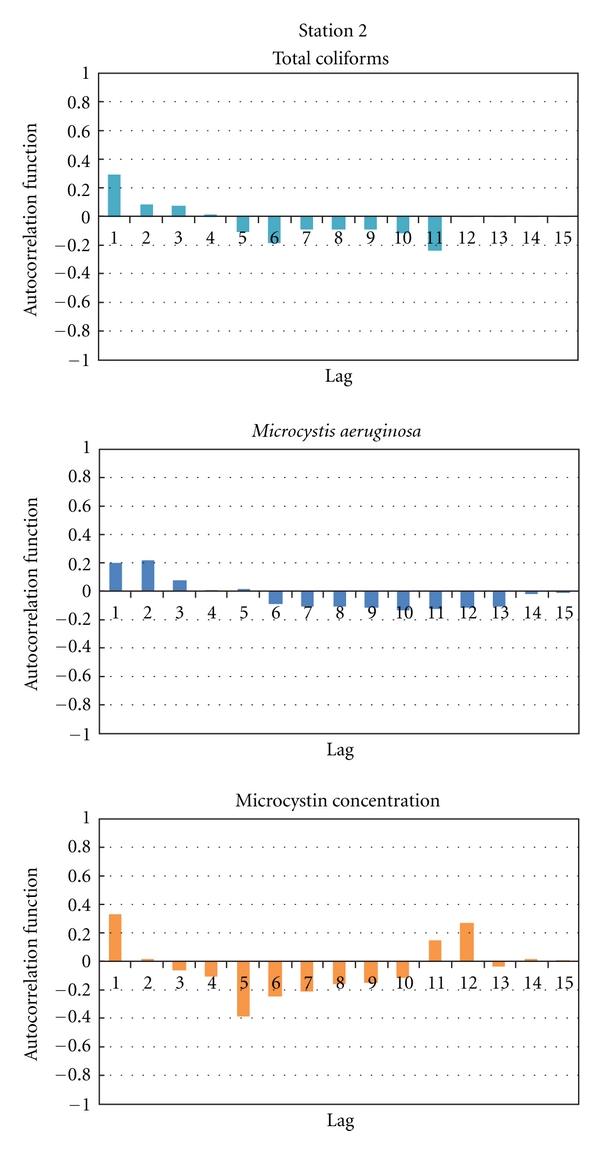
Examples of the autocorrelation functions (ACF) of coliforms, *M. aeruginosa,* and microcystin records of the water sampled at Station 2.

The autocorrelation function (ACF) was calculated for total coliforms, *Microcystis aeruginosa,* and microcystin concentration records from the 2004–2006 period. While the total coliforms and *M. aeruginosa* data did not exhibit a significant correlation for any lag or any discernible pattern, the microcystin records corroborated the results of the PCA and show some periodicity that confirmed the effect of environmental factors during the summer months. Examples of the ACF test are shown in Figure 5. Once the ACF test was performed, the data were used to produce histograms that allow us to assess the symmetry of counts' distribution and to select a parametric distribution function that describes them.

**Figure 6:**
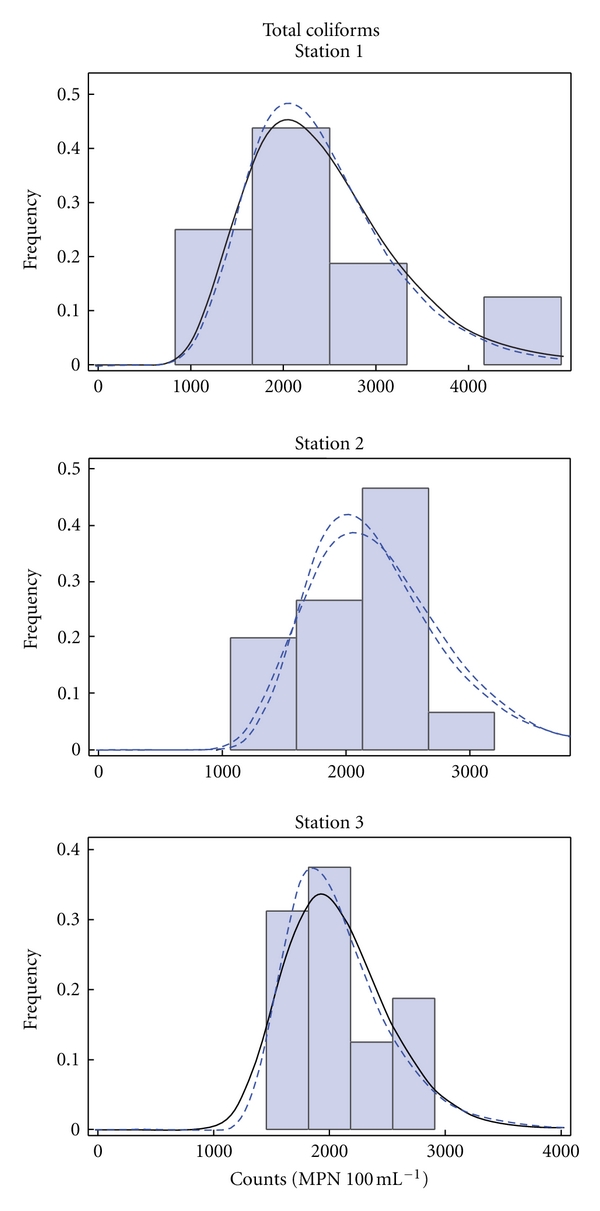
Histograms of total coliforms counts in the water at the three sampling sites described by the log-normal (solid line) and extreme value (dashed line) distribution functions.

**Figure 7:**
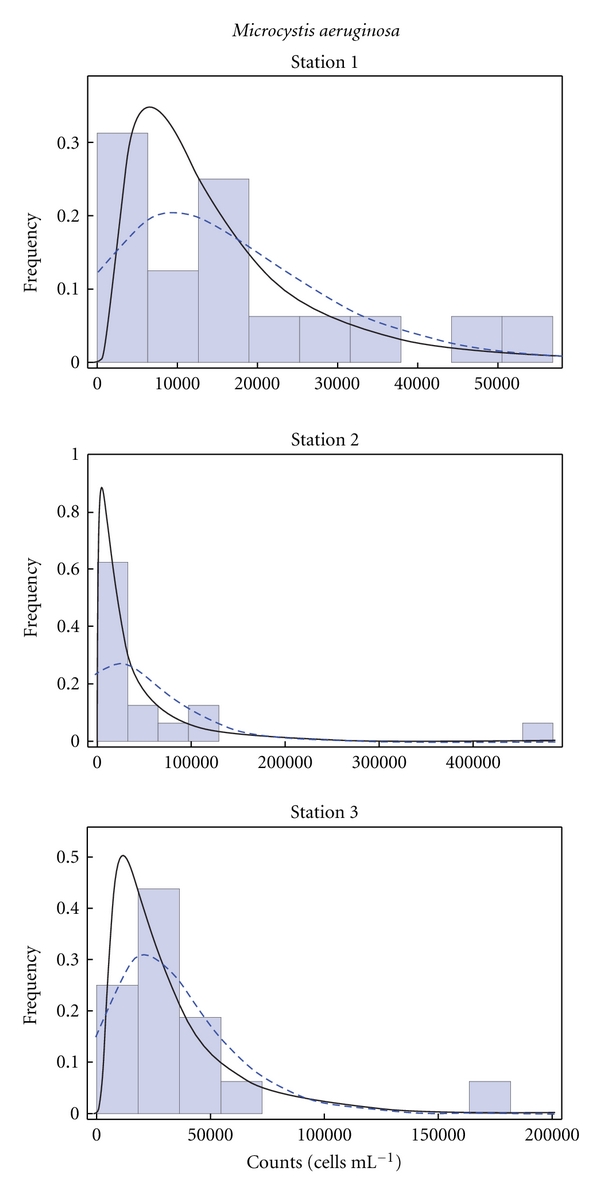
Histograms of *M. aeruginosa* cell counts in the water at the three sampling sites described by the log-normal (solid line) and extreme value (dashed line) distribution functions.

**Figure 8:**
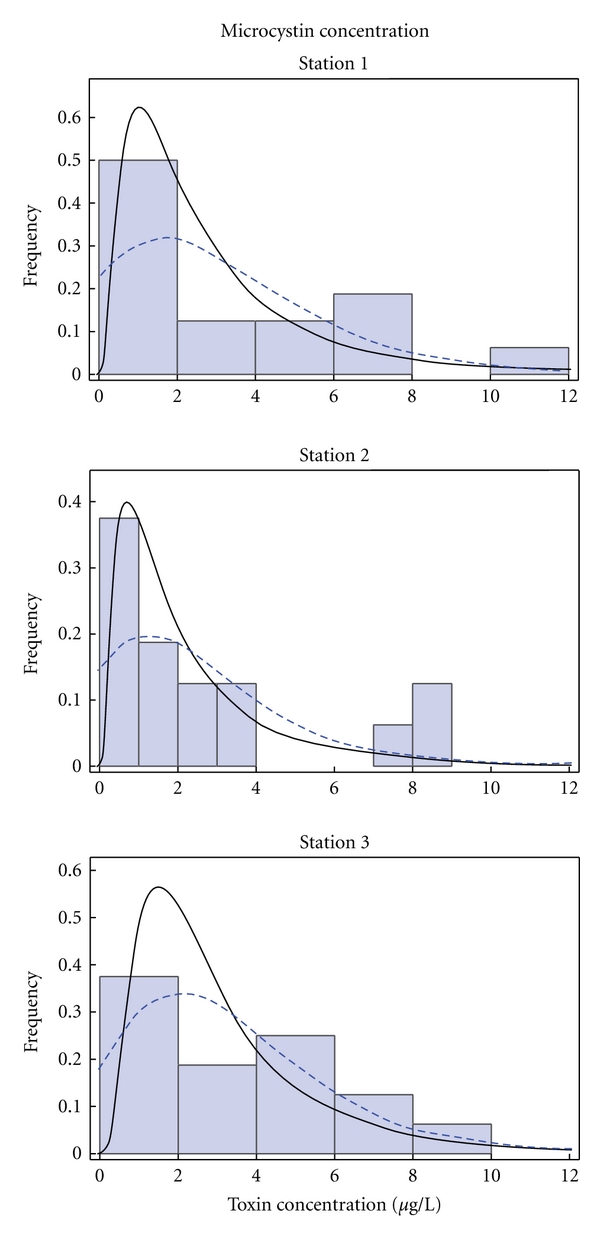
Histograms of toxin concentration in the water at the three sampling sites described by the Log-normal (solid line) and extreme value (dashed line) distribution functions.

The selection of appropriate distribution functions was based on the linearity of their corresponding Q-Q plots. For all records and sampling points, the log normal and the extreme value distribution functions were chosen. The histograms of the records of total coliforms, *M. aeruginosa,* and microcystin at the three sampling sites (Stations 1–3) described by the selected distribution functions are presented in Figures 6, 7, and 8, respectively.

The parameters of both distribution functions were estimated by the method of moments (MM) or through maximum likelihood estimation (MLE) as described in the methods section. Both methods resulted in similar estimates and neither had been consistently superior. The distribution's parameters for the total coliforms, *M. aeruginosa,* and microcystin records at the three sampling sites (Stations 1–3) are listed in Tables 2–4, respectively. From these data it is possible to calculate the probability that a count will exceed any given level, N_c_ [7]. The N_c_ values can be derived from current guidelines or regulations or they can correspond to levels that will require chemical treatment or a change in the water designated use. In the particular case of the Rio de la Plata estuary and since no local regulations are available, the reference cutoff values for total coliforms, *M. aeruginosa,* and microcystin were obtained from the guidelines of the European Union and the World Health Organization. The estimated numbers of counts exceeding two selected cutoff values, N_c_'s, calculated from the distributions derived from the available 2004–2006 records were compared with those observed in a new data set (i.e., the data collected during 2007). The comparisons are summarized in Tables 2, 3, and 4 for total coliforms, *M. aeruginosa* and microcystin concentrations, respectively. As observed in previous works [7, 37] the tables showed a reasonable agreement between the estimates and observations despite the uneven and sometimes low sampling rate and the fact that the data themselves had not always been perfectly independent. Minor violations of the assumptions on which this probabilistic approach is based do not render it inapplicable. However, they affect the predictions' reliability and reduce the estimates' accuracy. A transformation of the series of records as indicated in Corradini et al. [35] could reduce the effect of seasonality.

We are not proposing to use this probabilistic approach as a replacement of monitoring systems but to complement them and extract additional information from the data that they provide. In that sense, the estimated frequencies can be very useful to evaluate the water quality of a resource in terms of the probability that there will be microbial or toxic outbursts of safety concern. As pointed out by Hadas et al. [7] the possibility to estimate future high counts or concentrations enables assessing the water quality at the same site at different times of the year and evaluating the efficacy and/or risk on changes in treatments or sanitary measures.

## 6. Conclusions

The high total cyanobacteria, *Microcystis aeruginosa*, and total and feacal coliforms counts reflect the presence of anthropogenic pollution sources in the Rio de la Plata estuary. Waste water treatment does not constitute a generalized or extended practice related to urban development, agricultural, or industrial activities in Argentina. This shortfall leads to a progressive deterioration of watersheds such as the Rio de la Plata estuary.

Understanding the effect of environmental and anthropogenic factors on the production of microcystins could contribute to elucidate the mechanism involved in their biosynthesis as well as in the prevention of pollution leading to the dominance of toxic cyanobacterial blooms.

The microcystin concentrations observed in the Rio de la Plata estuary along this study are similar to those described from natural blooms worldwide [38–41]. MC-LR was the toxin most commonly found.

The probabilistic approach developed by Peleg et al. [42, 43] and applied in this study makes it possible to extract additional information from monitoring records and to evaluate sampling sites and conditions in terms of future microbial or toxic outbursts.

It should be mentioned that although the major route of human exposure to cyanobacterial toxins is the consumption of drinking water, a minor exposure route is the recreational use of lakes and Rivers [44]. Since the Rio de la Plata river fulfills both purposes, it is necessary to establish monitoring programs to prevent the presence of MC in drinking water, improve water treatment facilities to ensure the availability of safe sources of drinking water and avoid intoxication during recreational uses. Special attention should be directed to diminishing the degree of eutrophication of this water resource.

## Figures and Tables

**Table 1:** Table records of the biological, physical and chemical parameters for the three selected sampling points 1, 2, and 3.

Sample point 1													
Data sampling	*Microcystis aeruginosa *Cel·mL^−1^	Total cyanobacterial Cel·mL^−1^	T (°C)	pH	Conductivity *μ*S-cm^−1^	Total coliforms MNP·100 mL^−1^	Fecal coliforms MP·100 mL^−1^	Iron mg·L^−1^	Total phosphorus mg·L^−1^	Chlorophyll-a mg·L^−1^	Nitrates mg·L^−1^	Nitrites mg·L^−1^	Microcystin-LR *μ*g·L^−1^

12/03/2004	13200	16600	29.0	7.9	290	4500	4600	0.60	0.89	56	3.0	0.04	1.10
01/12/2005	7560	20040	23.5	7.9	295	4600	2400	0.50	0.80	108	3.3	0.06	4.21
02/03/2005	30000	31800	29.5	8.4	320	2400	2400	0.37	0.37	45	4.6	0.07	7.12
02/22/2005	32100	52800	24.0	8.3	304	2400	2400	0.34	0.40	55	5.3	0.08	2.52
03/11/2005	51000	51300	21.0	8.4	339	2400	2400	0.33	0.49	34	7.5	0.20	1.30
04/01/2005	48900	49800	20.0	9.6	273	1500	1500	0.40	0.50	23	7.0	0.25	1,10
04/19/2005	25000	40500	21.0	8.6	349	1500	1500	0.55	0.44	22	4.0	0.06	<0.1
06/02/2005	14850	17850	15.0	7.4	409	2100	2100	0.36	0.35	22	6.6	0.15	<0.1
08/01/2005	0	2700	12.0	8.1	353	1500	1500	0.30	0.20	15	5.6	0.06	<0.1
08/29/2005	0	4200	17.0	7.6	400	2100	2100	0.20	0.40	12	4.5	0.04	<0.1
10/20/2005	950	16600	22.0	7.4	314	2100	2100	0.46	0.51	23	4.6	0.05	<0.1
11/15/2005	4300	29300	21.0	7.7	299	1500	1500	0.46	0.45	33	12.0	0.06	2.22
12/01/2005	1500	27300	23.0	7.5	309	2300	1500	0.34	0.33	45	15.0	0.06	4.52
12/20/2005	10425	23400	30.0	7.8	310	2800	2300	0.27	0.43	56	3.6	0.06	7.81
01/09/2006	17100	23250	30.0	7.7	322	2800	2800	1.13	0.47	29	4.0	0.05	10.50
01/17/2006	14700	17700	29.0	7.8	310	2800	2300	0.20	0.83	55	3.6	0.06	7.73

Sample point 2													

Data sampling	*Microcystis aeruginosa *Cel·mL^−1^	Total cyanobacterial Cel·mL^−1^	T (°C)	pH	Conductivity *μ*S-cm^−1^	Total coliforms MNP·100mL^−1^	Fecal coliforms NNP·100mL^−1^	Iron mg·L^−1^	Total phosphorus mg·L^−1^	Chlorophyll-a mg·L^−1^	Nitrates mg·L^−1^	Nitrites mg·L^−1^	Microcystin-LR *μ*g·L^−1^

12/03/2004	101500	131180	23.0	8.2	379	4500	4500	0.48	0.33	55	3.8	0.06	3.30
01/12/2005	109897	148500	25.0	8.1	370	2800	2400	0.42	0.48	62	4.3	0.05	7.71
02/03/2005	458400	458400	24.0	7.3	389	2400	2400	0.20	0.43	66	4.6	0.02	0.56
02/22/2005	34200	34299	21.0	8.9	290	2400	2400	0.29	0.47	44	4.4	0.04	2.47
03/11/2005	66300	68100	20.0	8.3	379	2400	2400	0.30	0.38	55	6.0	0.07	1.46
04/01/2005	49200	49200	22.0	7.7	356	1500	1500	0.26	0.46	33	5.7	0.04	1.36
04/19/2005	20100	20100	16.5	7.6	352	1500	1500	0.43	0.25	30	2.8	0.02	<0.1
06/02/2005	150	150	14.0	7.6	379	2100	2100	0.79	0.45	29	6.2	0.04	<0.1
08/01/2005	750	750	11.0	8.0	350	2100	1500	0.35	0.24	28	6.1	0.04	<0.1
08/29/2005	500	500	20.5	8.5	345	2100	2100	0.42	0.11	22	6.2	0.06	<0.1
10/20/2005	6450	6450	17.0	7.8	350	2100	2100	0.21	1.00	21	11.0	0.04	<0.1
11/15/2005	7020	15360	28.0	8.5	273	1500	1500	0.48	1.24	58	4.5	0.03	2.20
12/01/2005	2580	17160	23.0	7.9	309	2300	1500	0.34	0.48	82	6.5	0.07	8.62
12/20/2005	6600	21000	30.0	7.8	285	2300	2300	0.44	0.45	32	2.3	0.06	8.12
01/09/2006	9600	14400	30.0	8.2	353	2300	2800	0.24	0.40	40	4.3	0.05	1.05
01/17/2006	16500	17700	29.5	8.2	350	2300	2300	0.34	0.25	44	4.4	0.06	3.25

Sample point 3													

Data sampling	*Microcystis aeruginosa *Cel·mL^−1^	Total cyanobacterial Cel·mL^−1^	T (°C)	pH	Conductivity *μ*S-cm^−1^	Total coliforms MNP·100 mL^−1^	Fecal coliforms NMP·100 mL^−1^	Iron mg·L^−1^	Total phosphorus mg·L^−1^	Chlorophyll-a mg·L^−1^	Nitrates mg·L^−1^	Nitrites mg·L^−1^	Microcystin-LR *μ*g·L^−1^

12/03/2004	56400	59800	23.0	7.6	310	2400	2400	0.49	0.31	34	5.6	0.04	4.43
01/12/2005	45800	67400	27.4	8.3	413	2100	2100	0.43	0.41	43	4.3	0.02	6.42
02/03/2005	34500	35100	27.4	8.3	413	2100	2100	0.43	0.41	53	4.3	0.01	5.52
02/22/2005	20850	30750	24.5	8.5	275	2100	2100	0.11	0.41	42	6.6	0.19	3.50
03/11/2005	31200	37350	21.5	10.2	435	1500	1500	0.26	0.35	52	7.4	0.03	2.05
04/01/2005	40500	40800	21.5	8.8	337	1500	1500	0.01	0.37	45	2.1	0.01	1.71
04/19/2005	5640	6840	18.0	8.5	356	1500	1500	0.03	0.45	23	3.3	0.06	<0.1
06/02/2005	0	1800	15.0	7.7	436	2100	1500	0.80	0.50	26	3.5	0.05	<0.1
08/01/2005	2050	2300	14.0	8.5	420	2300	2800	0.35	0.58	26	8.9	0.01	<0.1
08/29/2005	1550	2300	11.0	8.4	309	1500	2000	0.53	0.42	23	2.2	0.02	<0.1
10/20/2005	165000	175200	18.5	7.3	507	1500	1500	0.15	0.05	20	1.3	0.07	<0.1
11/15/2005	27000	31500	23.0	7.7	407	2800	2800	0.63	0.07	56	1.4	0.07	3.51
12/01/2005	29100	107700	26.0	8.1	306	2100	2300	0.61	0.37	45	1.9	0.06	7.80
12/20/2005	33300	176100	23.0	7.4	445	2100	2100	0.32	0.40	54	7.2	0.09	8.11
01/09/2006	28800	30000	25.0	7.8	435	2800	2100	0.43	0.45	44	6.8	0.06	5.33
01/17/2006	44100	56400	27.0	7.8	452	2800	2100	0.44	0.77	34	5.9	0.07	4.50

**Table 2:** Parameters, goodness of fit measure and estimated probability of total coliforms above specified values for each distribution function versus observed frequencies at each sampling site.

Sampling site	Distribution functions	Dist. parameters		Goodness of fit	Estimated counts above N_c_ from 2004 to 2006 data versus			
					observed data 2007			
		*α*	*β*	*χ* ^2^	N_c_≤ 2000 MPN 100 mL^−1^		N_c_≤ 2200 MPN 100 mL^−1^	
					Estimated	Observed	Estimated	Observed
Station 1	Log-normal	7.8	0.3	7.6	10-11	5	8-9	3
	Extreme value	2050	710	7.6	10-11		8-9	
Station 2	Log-normal	7.7	0.3	6.8	9-10	10	7-8	8
	Extreme value	1990	520	17.3	9-10		7-8	
Station 3	Log-normal	7.6	0.2	16.4	7-8	4	5-6	3
	Extreme value	1870	360	16.4	7-8		5-6	

**Table 3:** Parameters, goodness of fit measure and estimated frequency of *Microcystis aeruginosa* above specified values for each distribution function versus observed frequencies at each sampling site.

Sampling site	Distribution functions	Dist. parameters		Goodness of fit	Estimated counts above N_c_ from 2004–2006 data versus			
					observed data 2007			
		*α*	*β*	*χ* ^2^	N_c_≤ 15000 cells mL^−1^		N_c_≤ 20000 cells mL^−1^	
					Estimated	Observed	Estimated	Observed
Station 1	Log-normal	9.4	0.8	7.6	6-7	5	4-5	5
	Extreme value	9850	11430	3.3	7-8		5-6	
Station 2	Log-normal	10.1	1.3	5.9	9-10	9	8-9	9
	Extreme value	21470	43600	18.1	10-11		9-10	
Station 3	Log-normal	10.1	0.9	11.1	10-11	9	8-9	9
	Extreme value	21440	21600	8.5	11-12		9-10	

**Table 4:** Parameters, goodness of fit and estimated probability of microcystin concentration above specified values for each distribution function versus observed frequencies at each sampling site.

Sampling site	Distribution functions	Dist. parameters		Goodness of fit	Estimated counts above N_c_ from 2004–2006 data versus			
					observed data 2007			
		*α*	*β*	*χ* ^2^	N_c_≤ 4 *μ*g L^−1^		N_c_≤ 5 *μ*g L^−1^	
					Estimated	Observed	Estimated	Observed
Station 1	Log-normal	7.7	0.3	15.5	3-4	0	2-3	0
	Extreme value	1910	490	15.5	4-5		3-4	
Station 2	Log-normal	7.7	0.3	15.5	2-3	2	2-3	2
	Extreme value	1910	480	15.5	3-4		1-2	
Station 3	Log-normal	7.6	0.3	15.5	3-4	0	2-3	0
	Extreme value	1840	330	15.5	4-5		3-4	
